# Insights from the Molecular dynamics simulation of BcsD Subunit from K. xylinus

**DOI:** 10.6026/97320630013376

**Published:** 2017-11-30

**Authors:** Simranjit Kaur, Mahesh Kulharia

**Affiliations:** 1 Centre for Human Genetics and Molecular Medicine, School of Health Sciences, Central University of Punjab, Bathinda - 151001, India;; 2 Centre for Computational Sciences, School of Basic and Applied Sciences, Central University of Punjab, Bathinda - 151001, India;

**Keywords:** AHL's, Bcs, Homology Modeling, Bacterial Biofilms

## Abstract

Biofilms are bacteria living in micro-colonies with a protective coating in sessile form. The biofilm protects bacteria from harsh surroundings as well as help in antibiotics resistance using a semi-fluid substance. Cellulose is the major component of biofilm, which provides the sticky appearance to bacteria for attaching to the substratum. The bacteria communicate in biofilm with the help of quorum sensing hormones Acylated Homoserine Lactones (AHL's). In Komagataeibacter xylinus the four genes Bcs A, Bcs B, Bcs C, Bcs D are associated with cellulose biosynthesis. The Bcs D subunits have a hypothetical octamer pore-like structure through which glucan molecule pass to form the cellulose. Therefore, it is of interest to document a structural understanding of Bcs D. Hence a homology model of Bcs D was simulated and analyzed further to gain functional insight towards biofilm formation.

## Background

Biofilms are explained as a living thing in a thin layer [1]. Biofilms, secreted by bacteria enable it to behave in a group and become associated with each other in an aggregation [2]. Bacteria can aggregate together in a biofilm, e.g. in the mouth of animals [3]. Bacterial biofilms are less susceptible to most of the antimicrobial agents and antibiotics [4] and become resistant to host's immune system [5]. The less susceptibility of bacterial biofilms is due to a difficulty in penetration of antimicrobial agents through the biofilms. The polymers of biofilms resist the penetration of antimicrobial agents in the biofilms [6]. Biofilms were secreted by various bacterial species gram-positive bacteria such as Bacillus sp., Staphylococcus sp., gram-negative bacteria such as Escherichia coli, Pseudomonas sp. [7]. Bacteria also colonizes in plants such as Pseudomonas sp., and nitrogen-fixing bacteria such as Rhizobium leguminosarum Sinorhizobium meliloti, etc. [8]. Biofilms formation also detected in the animal body, for example, many bacterial species coagulate with their specific partners in the mouth of animals [3] and sometimes with multiple partner species and these species in turn aggregate with many other partner species. Multiple aggregations lead to the formation of thick plaque. The study of biofilms because necessary due to dispersion property of biofilms. It consists of channels through which the nutrition and secretions of the cell circulate [9]. Cells of bacterial biofilm express a distinct pattern of gene expression. Hence, different parts of biofilms are formed in a single aggregation of the bacterial colony [10]. The complexity of structure and function make it cognate of tissues of higher organisms [4]. Cellulose is a polysaccharide found in plenty. It is produced by both plants and bacteria. Bacterial cellulose formation and its role have been described in various bacterial species such as Gluconobacter xylinus, Sarcina ventriculi, E.coli, etc. [11]. Sequence analysis shows that various other bacteria such as Vibrio, Yersinia, etc. have also the ability to synthesize cellulose. G.xylinus was grown in vitro and a thick cellulose containing biofilms [12]. The cellulose was synthesized in a long polymeric chain and composed of β-1-4 linked D-glucose units. The Dglucose units were linked to a multimeric enzyme complex in the cytoplasm. A pore-like structure was located at the outer membrane for cellulose synthesis [12]. The genes responsible for cellulose synthesis were located in an operon. The four genes Bcs A, Bcs B, Bcs C, Bcs D are involved in biofilm formation. The transmembrane domains attach the Bcs A enzyme in cytoplasmic membrane and Bcs B protein in spatial proximity with Bcs A. The second messenger c-di-GMP has released by the activity of Bcs B which in turns activates Bcs A enzyme. The c-di-GMP (PilZ domain) was predicted at the C-terminal end of Bcs A cellulose synthase [13]. The functions of Bcs C and Bcs D are unknown. Saxena et al. (1994) reported that the Acs D gene of Acs operon of Acetobacter xylinus is not necessary for cellulose synthesis but is necessary for the standard rate of cellulose-I microfibrils synthesis [14]. The structure of Acs D protein with cylindrical octamer in periplasm that interacts with Cellulose Complementing Complex Ax (Ccp Ax) isolated. The Acs D has interior space that may act as a channel for nascent glucan chain. It supports the concept for pore complex. Similarly, Bcs D and Bcs H (Cap A) seem to be required for the Bcs complex formation in K.xylinus. It has indicated that the Bcs D has octamer pore-like structure that may help the glucan molecules to pass through the pore. Glucan molecule is essential for cellulose synthesis [15].

## Methodology

The study was conducted using Intel(R) Core (TM) i5-4200U CPU @1.60 GHz and 64-bit operating system.

### Alignment of query and template sequences

The Query sequence is the FASTA format of amino acid sequence with unknown protein structure was obtained from UniProt database with Entry-P19451, entry name-BcsD1_KOMXY, Gene name-BcsD, organism K.xylinus (Gluconacetobacter xylinus), length- 156, C's). The protein structure of Bcs D was not known, further confirmed from PDB databank.

Template protein structures refer to the sequence of the protein that resembles the query sequence to maximum identity and template sequence is used to model the structure of the query sequence. FASTA sequence was obtained from UniProt. Maximum identity was confirmed in the Basic Local Alignment Search Tool (BLAST) database. The 2 sequences producing significant alignments were obtained with e-value better than threshold- PDB: 3A8E and PDB: 3AJ1_A Emboss_needle program was used for the alignment of both the query and template sequences. The Sequence with PDB id 3AJ1_A was selected for as a template with 75% percent identity.

### Modelling the protein structure

MODELLER9.15 is homology-modeling software used to model protein structure from amino acid sequence. It creates an atomic resolution model of the target protein. The model of the target protein is produced from the amino acid sequence of a protein and 3D structure of the related homologous protein [14]. The quality and excellence of the target structure i.e. homology model depend on status and standard of DOPE Score Discrete Optimized Protein Energy (DOPE) a statistical energy potential. It accounts for shape and finite size of proteins. DOPE score gives a score to predicted models by considering positions of nonhydrogen atoms. Lower the DOPE score, more accurate is the predicted model as well as GA341 criterion score composite foldassessment score. It joins Z-score of potential statistical function, sequence similarity between target and template, and structural compactness. It ranges from 0.0 to 1.0. Models with a GA341 score higher than 0.7 have correct protein folding.

A PDB file was generated using Modeller and that is used for the simulation of the newly constructed model, with Gromacs. In Gromacs simulation firstly the topology was prepared and the solvated box was defined, then after adding ions energy was minimized and the system was equilibrated and with production Molecular Dynamics (MD) data were collected and analyzed.

## Result and Discussion

Bacteria adapts by synthesizing the coating of cellulose around sessile cells. The various species of bacteria are able to survive in an environment surrounded by cellulose. They use various mechanisms to resist biocides and antimicrobial agents. Therefore, it is of interest to develop and analyze a structural model of Bcs D protein. Bacteria deep inside of biofilm usually lack nutrition with in slow growth having resistance to biocides. The survival strategies of biofilm bacteria have an advantage over anti-microbial agents. The dispersion of developed biofilm plays a role in steadily spreading such bacteria. The operon consisting of four major genes Bcs ABCD are involved in biofilm formation in K. xylinus. The structure of Bcs A and Bcs B complex was identified and isolated from Rhodobacter sphaeroides. However, the molecular function of Bcs C and Bcs D is unknown. It has been known that Bcs D has octamer pore structure to help the glucan molecules to pass through it and is important for cellulose synthesis. It arranges cell complex along the longitudinal axis. It is also known that the Cellulose Complementing Factor CcpA (Bcs H) is required for Bcs activity in K. xylinus and K. laserii.

**Figure 1:**
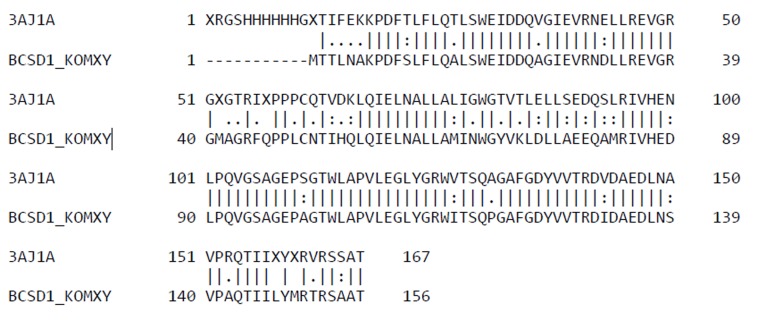
Alignment result of query sequence (BCSD1_KOMXY) against target sequence (3AJ1A) showing 70.1% identity and 79.6% similarity.

**Figure 2:**
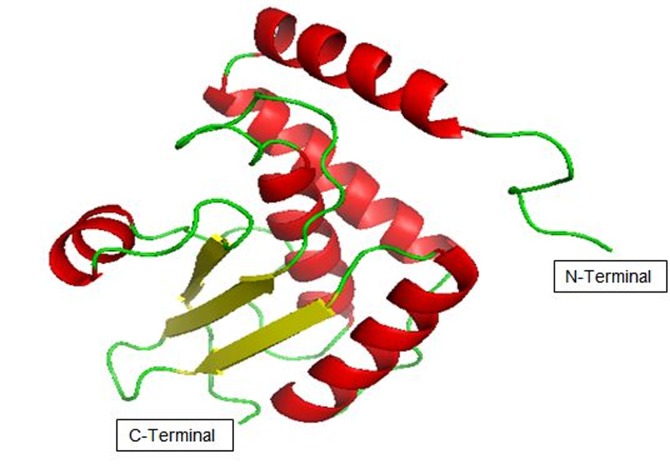
Structure of Bcs D protein model (156 residues long) from k. xylinus. This model is generated using the MODELLER9.15. This image is generated using PYMOL.

**Figure 3:**
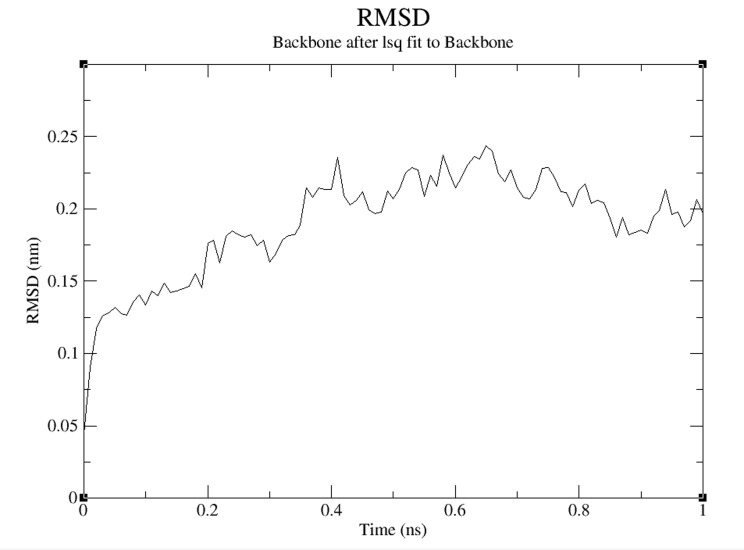
RMSD backbone plot (A) of Bcs D model during molecular dynamics simulation in GROMACS force field over ns time period. In the RMSD backbone plot, the RMSD value should be ~0.1nm. It indicates the structure is stable if any change between plots indicates the structure is slightly different from its crystal structure.

**Figure 4:**
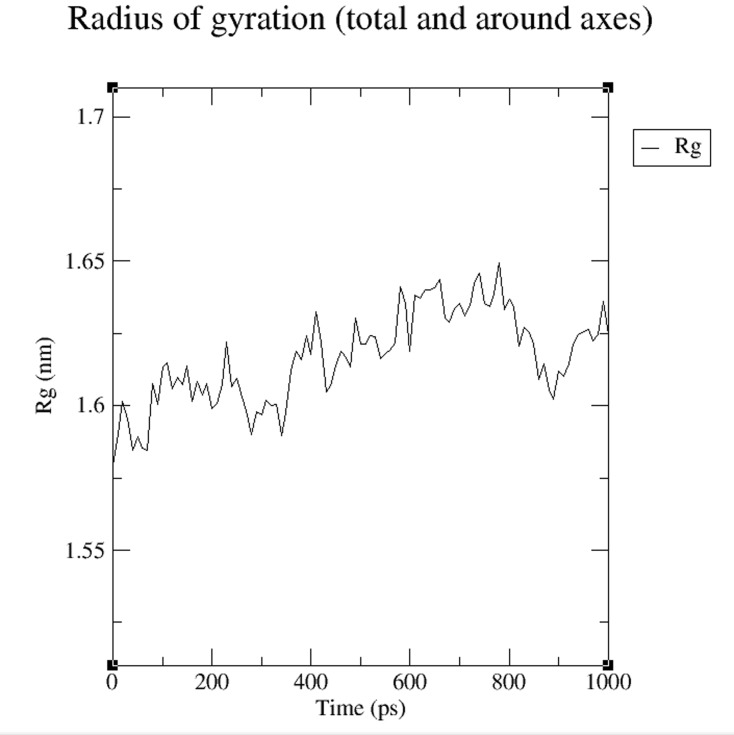
Radius of gyration plot of Bcs D model during molecular dynamics simulation over 1000 ps time period. In the RMSD gyration (Rg) plots, the radius of gyration analyzed (a measure of compactness of protein) during simulation.

The structure model of Bcs D protein consists of five α helices (red), 3 β-sheets (yellow) and 9 Random coils (green) as shown in Figure 2. This model was generated using homology modeling with MODELLER. An alignment of template and Bcs D query sequences was completed using EMBOSS Needle as shown in Figure 1. The RMSD deviation of the simulated model in GROMACS force field 1000 ps is shown in Figure 3. It is noted that the RMSD value lies within 2.5 Å (accepted range) over the simulation period. Radius of gyration plots of Bcs D model during molecular dynamics simulation over 1000 ps time period is shown in Figure 4. The plot in Figure 4 illustrates that the Rg value of protein structure model at a temperature of about 300K remains invariant. The accessible surface area (ASA) of the protein model was calculated with probe radius 1.4 Å. The protein model consists of 761 surface atoms and 444 buried atoms. The total area/energy is 9797.72 with 6155.81 apolar area/energy and 3641.9 polar area/energy. These data have important role in the understanding of its molecular function in the development of protective bio-films.

## Conclusion

A simulated model of Bcs D subunit of K.xylinus is reported in this study. The structure model consists of 3 beta sheets, 5 alpha helixes, and 9 random coils. The RMSD and Rg profile of the model during simulation in GROMACS force field is documented to gain molecular insight into its function in biofilm formation. It is also of further interest to study its interaction with other subunits in this context
